# Beyond patient classification: the “hidden” face of nursing workload

**DOI:** 10.1590/1980-220X-REEUSP-2021-0533en

**Published:** 2022-06-17

**Authors:** João Lucas Campos de Oliveira, Danielle Fabiana Cucolo, Ana Maria Müller de Magalhães, Marcia Galan Perroca

**Affiliations:** 1 Universidade Federal do Rio Grande do Sul, Nursing School, Graduate Program in Nursing, Porto Alegre, RS, Brazil.; 2 Pontifícia Universidade Católica de Campinas, Campinas, SP, Brazil.; 3 Faculdade de Medicina de Rio Preto, Programa de Pós-Graduação em Enfermagem, Rio Preto, SP, Brazil.

**Keywords:** Workload, Personnel Downsizing, Nursing Assessment, Nursing Staff, Hospital, Hospital Administration, Carga de Trabajo, Reducción de Personal, Evaluación en Enfermería, Personal de Enfermería en Hospital, Administración Hospitalaria, Carga de Trabalho, Dimensionamento de Pessoal, Avaliação em Enfermagem, Recursos Humanos de Enfermagem no Hospital, Gestão Hospitalar

## Abstract

This is a theoretical-reflective essay, which aimed to reflect on the centralization of Patient Classification Systems in workload and hospital nursing staff sizing. The reflexive interpretations were guided by two axes: *Patient Classification Systems : constitution and utilities*; and *“Hidden” nursing activities in workload measurement*. The first evidences the importance and the role of these instruments in workload identification and in hospital nursing staff sizing, exemplifying several possibilities to be used by nurses. On the other hand, with the second axis, it is clear that there are many nursing activities that are not sensitive to the application (even if systematic) of these means of patient assessment. Therefore, nursing workload measurement may be underestimated. It was inferred that the complexity of practice environments requires a macro and micro institutional look at the nursing workload measurement, especially when considered for workforce planning/sizing purposes.

## RESUMO

Ensaio teórico-reflexivo, que objetivou refletir acerca da centralização dos Sistemas de Classificação de Pacientes na aferição da carga de trabalho e dimensionamento de pessoal de enfermagem hospitalar. As interpretações reflexivas foram norteadas por dois eixos: *Sistemas de Classificação de Pacientes: constituição e utilidades*; e *Atividades de enfermagem “ocultas” na aferição da carga de trabalho*. O primeiro evidencia a importância e o protagonismo desses instrumentos na identificação da carga de trabalho e no dimensionamento de pessoal de enfermagem hospitalar, exemplificando-se diversas possibilidades a serem utilizadas pelos enfermeiros. Em contrapartida, com o segundo eixo, percebe-se que existem muitas atividades de enfermagem que não são sensíveis à aplicação (mesmo que sistemática) destes meios de avaliação do paciente. Logo, a mensuração da carga de trabalho da enfermagem pode ser subestimada. Inferiu-se que a complexidade dos ambientes de prática requer um olhar macro e micro institucional à aferição da carga de trabalho da enfermagem, especialmente quando considerada para fins de planejamento/dimensionamento da força de trabalho.

## RESUMEN

Ensayo teórico-reflexivo, que tuvo como objetivo reflexionar sobre la centralización de los Sistemas de Clasificación de Pacientes en la evaluación de la carga de trabajo y dimensionamiento del personal de enfermería hospitalario. Las interpretaciones reflexivas fueron guiadas por dos ejes: Sistemas de Clasificación de Pacientes: constitución y utilidades; y Actividades de enfermería “ocultas” en la medición de la carga de trabajo. El primero evidencia la importancia y el papel de estos instrumentos en la identificación de la carga de trabajo y en el dimensionamiento del personal de enfermería hospitalario, ejemplificando varias posibilidades para ser utilizados por los enfermeros. Por otro lado, con el segundo eje, es claro que hay muchas actividades de enfermería que no son sensibles a la aplicación (aunque sistemática) de estos medios de evaluación del paciente. Por lo tanto, la medición de la carga de trabajo de enfermería puede estar subestimada. Se infirió que la complejidad de los ambientes de práctica exige una mirada macro y micro institucional en la medición de la carga de trabajo de enfermería, especialmente cuando se considera para fines de planificación/dimensionamiento de la fuerza de trabajo.

## INTRODUCTION

The hospital dynamics imposes on the nursing team some peculiar characteristics of work organization and management, including the division of interconnected shifts, uninterrupted care and staff allocation, in accordance with hospitalized patients’ demands and hospital sectors’ needs. In work organization, it is common – and necessary – for nurses to use means and instruments for distributing and managing tasks to the led team, in order to enable comprehensive, qualified and safe care^(1)^.

It has been noticed that the care complexity of hospital users is exponential, both due to the advance and sophistication of diagnostic-therapeutic interventions and the population demographic and epidemiological transition, with a focus on aging and high incidence of chronic diseases, in addition to increase in injuries from external causes^(2)^. The nursing team activities also accompany this growing complexity, while attributions are constantly added to meet the high demand for care, highlighting nursing workload (NWL) as a major issue in the arena of health service management debates in different socioeconomic scenarios^(3–6)^.

The concept of workload in the nursing area is complex, dynamic and even polysemic. A study^(5)^ of conceptual analysis conducted by a researcher based in California, United States of America, identified that it would be related to five elements: nursing time; professional competence level; weight/impact of direct patient care; amount of physical effort demanded; and customer care complexity. In this way, NWL was defined as the amount of time and care that a nursing worker can dedicate (directly and indirectly) to patients, the workplace and professional development^(5)^.

Imbued with the concept of workload, the time variable is central to the assumptions related to the forecast of nursing staff^(6)^. In hospital units, the measurement of direct and indirect care time can be performed by Patient Classification Systems (PCS), since, by determining a category/level of care dependence through these instruments, it is possible to estimate the expected nursing time, per patient, in 24 hours^(1,7,8)^. PCS correspond to a systematic way of assessing patients under some aspects/areas of interest to nursing care and work, attributing a judgment of nurses about each one of them and, consequently, framing patients in a certain gradation of complexity.

When estimating the time of nursing care, PCS become a protagonist tool in the processes of staff sizing in the hospital environment^(6)^. Moreover, it enables the planning of direct daily care, contributing to costs estimation and management. In contrast, there are activities that demand nursing time and that go beyond the sensitivity of PCS assessment; therefore, they deserve to be considered in the workforce planning process, even if this is not verified in practice^(9)^.

Considering the positive/negative ambivalence that the (in)adequacy of nursing staff has on quality and safety results in patient care^(3,4)^, that the use of PCS is a consolidated means in the process of sizing hospital nursing staff and that there is a need for further discussions regarding the nursing activities considered in this process, this study aimed to reflect on the centralization of PCS in measuring workload and hospital nursing staff sizing.

## METHOD

This is a theoretical-reflective study. It was developed by researchers interested in the themes of workload and nursing staff sizing, based in the Brazilian states of Rio Grande do Sul and São Paulo.

The reflection was guided by national and international technical-scientific support regarding PCS, elements for measuring NWL and staff sizing, in addition to the authorial experience, which are duly exposed throughout the text linked to the proposed reflection. For organizational purposes, the reflection was guided by two axes, which include interpretive inferences and are described below.

### Patient Classification Systems: Constitution and Utilities

The first studies on PCS appeared more than 40 years ago and, since then, the instruments have been improved, and their use in nursing practice has been consolidated^(7,8,10)^. By revealing patients’ needs in relation to nursing care, this tool has helped in workload management, in order to balance available resources and clinical demands, and also support workforce planning^(10)^, even referred to as the basic element in this process^(6)^.

In the United Kingdom, dilemmas related to cost containment, shortage of nursing staff and variability in the occupancy of hospital units led to the proposition of a flexible model of allocation of professionals guided, above all, by patient classification^(11)^. In this proposal, an effective staff is recommended to meet up to 90% of the time required by patients, adjusted by a floating team with competence compatible with the demands of care and/or temporary hiring, when necessary^(11)^, making up determining factors in the cost-effectiveness of work production and in quality of care.

In addition to contributing to decision-making at the sectoral and institutional levels, considering the clinical judgment of nurses performed at the bedside, PCS can be used to establish priorities for patient care and provide interventions, constituting an important instrument for the nursing process^(1,12)^, i.e., care management.

It is important to emphasize that, in line with best practices, the choice of valid and reliable instruments generates greater certainty about the results obtained^(1,10)^. The validity and reliability of a PCS are important to extract precisely what is intended to be assessed, but also because the results of this assessment have repercussions on a concrete projection of human capital for care production, in addition to supporting direct care planning. In clear words, an imprecise, incomplete and/or invalid PCS may not only misdetermine the number and qualification of nursing workforce, but also may not actually contribute to care management by nurses at the bedside. Thus, it is postulated the need for a broader and scientifically well-founded look at PCS, which is the domain of nurses and supports them both in care management, based on the assessment/detection of patient demands, and in the rational workforce distribution.

If used systematically, in addition to professional (NWL measurement and staffing/distribution) and care (identification of needs and prioritization of care) dimensions, PCS can help leaders in forecasting structural, technological, educational investments and in interprofessional practice. This means that the recognition of the complexity of patients assisted in each sector can be a parameter for the budget (re)planning of equipment/materials and technologies, in addition to the number of personnel.

Team development can also be (re)organized by changing the profile of the level of dependency of patients treated and/or verifying more representative areas of care. In practice, for example, if the area of assessment of a PCS related to mobility and ambulation is very representative, therefore, the integration of other professionals into the unit’s workforce (such as the physical therapist, for example) can be rethought, emerging as a proposal for articulating actions and collaboration in health care.

At the national level, the Federal Nursing Council^(12)^ establishes minimum parameters for staffing in inpatient units (IU), among others. All requirements (nursing/patient hours in 24 hours, proportion of professionals/patient and percentage distribution of total professionals in categories) are related to the level of care dependency defined by a PCS. This resolution explicitly indicates PCS for adult^(1,7)^ and pediatric^(8)^ IU clientele, in addition to mental health related^(13)^.

PCS developed in Brazil are composed of critical indicators/areas of care and graduated, increasingly, in terms of care complexity and/or level of patient dependence. In practice, they can be understood as scales; therefore, the sum of the points of indicators/areas allows stratifying patients in categories or strata/levels of complexity^(1,7,8,14–16)^.

The Fugulin patient classification instrument^(7)^ was developed in 1994, and, after modifications, it came to consist of nine areas of care (mental status, oxygenation, vital signs, motility, ambulation, feeding, body care, elimination, and therapy), graded from 1 to 4 points. The final score categorizes patients into: minimal care – from 9 to 14 points; intermediate care – from 15 to 20 points; high dependence care – from 21 to 26 points; semi-intensive care – from 27 to 31 points; intensive care – above 31 points. It is worth noting that this categorization is the one contained in the current Brazilian regulations on nursing staff sizing, when it comes to the personnel sizing mediated by PCS^(12)^.

A PCS elaborated and validated by Perroca was reformulated, and its new version had its psychometric properties tested^(1)^. In it, nurses’ opinions about their use and activities related to care management were incorporated and represented in nine areas of care: care planning and coordination; investigation and monitoring; personal hygiene and eliminations; skin integrity; nutrition and hydration; locomotion or activity; therapeutic, emotional; support and aid; and health education. Patients must be scored (1 to 4) in each area and, at the end, will be stratified into the following categories: minimal care (9 to 12); intermediate care (13 to 18); semi-intensive care (19 to 24); and intensive care (25 to 36 points)^(1)^.

In pediatric care management^(8)^, the Pediatric Patient Classification Instrument (PPCI) groups areas of care by domains: family (companion participation and family support and support network); patient (activity, oxygenation, mobility and ambulation, food and hydration, eliminations and hygiene and body care); and therapeutic procedures (measurement interval and controls, drug therapy and cutaneous-mucosal integrity). The scores for each assessment item also vary from 1 to 4 points, with five categories: minimal care (11–17); intermediate care (18–23); high dependency care (24–30); semi-intensive care (31–36); and intensive care (37–44).

In addition to the PCS mentioned, recent national studies mention others for specialized units, such as neonatology^(14)^, rooming-in (maternity)^(15)^, in addition to an update^(16)^ of the PCS cited in the current regulations as the one recommended for psychiatric clients^(13)^. It is believed that this movement of expansion, updating and plurality of PCS is natural, expected and healthy, even because the spaces of nursing practice are undeniably multiple and, therefore, the needs for clientele and NWL assessment are equally diverse. However, this does not contradict the need for strict compliance with the assumptions of validity and reliability already problematized in the strategic definition of a PCS and/or other means of measuring NWL.

In addition to the Brazilian reality, there are instruments used in other countries, such as Finland, Norway and Canada^(17,18,19)^, such as computerized systems, which have the potential to contribute to government decisions on health. That is, in possession of this information, the government assesses the costs of nursing care and incorporates this analysis into the financing of services.

Although it is not a PCS, as it does not stratify patients in a level/stratum, but determines a score/score, the Nursing Activities Score (NAS) has been widely used worldwide as a systematic means of measuring NWL in Intensive Care Units (ICU). This allusion is confirmed by recent research findings that aimed to: investigate the relationship between workload and nursing mental burdens in Iran^(20)^; assess costs in Brazil^(21)^; verify clinical outcomes among critically ill patients in Greece^(22)^; and compare the intensive care NWL from the perspective of the COVID-19 pandemic in the Netherlands^(23)^.

The NAS, of North American origin, was adapted and validated for the Brazilian culture^(24)^, determining the time that nursing should dedicate to patients within 24 hours. Organized into seven categories (basic activities, ventilatory support, cardiological, renal, neurological, metabolic and specific interventions) it has 23 items to be scored by nurses equivalent to care needs. As shown, the instrument does not categorize patients into levels of complexity, but generates a total score, representing the percentage of time spent, per shift, for direct patient care^(20–24)^. By transforming the NAS score into time, it is considered fully possible to make nursing staff sizing feasible as predicted by the use of a PCS, so, and also considering its global scope, it is suggested that it be explicitly incorporated into the regulation on Brazilian nursing staff sizing.

The daily stratification of patients requires time, clinical competence, and does not exempt nurses from recording their assessments and interventions in medical records, as they are complementary activities. Nurses perform multiple tasks during the work shift, which creates the need to prioritize activities, in addition, often due to time constraints and, also, for not participating in the discussions/decisions on personnel sizing, it does not recognize the value of this practice^(17,19)^. These allusions reinforce the importance of team involvement in NWL management and also of clear knowledge about the real purpose and applicability of PCS. In other words, it is necessary for the actors who operate the daily classification of patients to recognize the importance of this practice and the purposes for which it is institutionalized.

Another critical aspect in the data interpretation obtained refers to the use of the average time of patients without considering the individual variation (of patient and/or professional) to complete a task^(25)^, which must be considered in negotiations and workforce planning. So, given the diversity of instruments available to stratify patients, English authors^(25)^ recommend greater ownership by nurses/managers of the different ways to apply them, in addition to scientific production on the necessary investments and better incorporation of the results obtained.

PCS are not administrative instruments and should not fit into a bureaucratized practice. It is necessary to recognize, however, the diversity of forms, protocols and scales implemented in institutions under the responsibility of nurses. These demands can imprint a work dynamic loaded with evaluative actions, but not very interpretive/resolving. In addition, it is considered premature and even innocent to believe that a single scale/ instrument can accurately measure the completeness of the nursing work demand.

### “Hidden” Nursing Activities in Workload Measurement

In the last decade, researchers^(3–5,25–29)^ have advanced in NWL investigation, including factors not included in PCS. Nurses’ professional experience, for instance, can influence the time dedicated to patient admission, regardless of the level of complexity, since the agility and quality of this activity can be favored by the skills acquired with the accumulation of experience and professional development^(27)^.

Another aspect to be noted refers to interaction with other professionals. Nursing does not develop its work in isolation, and team conformation influences the fulfillment of each unit’s demands^(9,25)^. Communication between professionals, support services and via telephone, in addition to travel, both within and outside the sector, consume significant time for nurses^(28)^, especially when there is no specific team to transport patients, and this is not directly verified in a PCS, at least among those used at the national level. This means that, in addition to verifying the time dedicated to care with and for patients, the profile of the nursing team and collaboration between professionals/services, including interactions between client and provider, need to be considered in NWL measurement and management.

The limits of PCS also involve actions not related to patients, such as development and supervision of personnel, conflict resolution, control of materials/equipment, reporting of incidents/adverse events, among others. However, it would be difficult to objectively assess all these and other activities inherent to nursing work in a single instrument. Thus, PCS are, admittedly, valuable tools for nurses and managers to identify part of a more comprehensive and complex construct that is NWL, and which walks in the field of research, but needs to be better appropriated in practice scenarios.

Nurses’ perception of the intensity of daily work can be a first step, as it comprises a critical indicator of NWL^(26)^. A study carried out in Brazilian hospitals, considering this judgment, showed four indirect care interventions: preceptorship of employees (support/guidance to newly hired or transferred); team development; conflict mediation; and support to doctors^(29)^. According to the authors^(29)^, in private hospitals, support actions (to the medical professional and to the new professionals) demanded more time from nurses, while, in educational institutions, the verification/control of medicines, the environment and laboratory data was more expressive.

The aforementioned findings reinforce the need to manage the NWL beyond the clinical complexity of patients, because, although some instruments allow exploring indirect care activities, nurses’ role is very comprehensive within a unit, and the focus of their practice may be different in each health organization, including hospitals. An example of this is the level of ownership of the Nursing Process in a unit, because, although it is an activity strictly related to direct care planning, it is not considered in the measurement of nursing time in all PCS. Another example is the application of other care management instruments, such as risk assessment scales for falls and pressure injuries, common in hospital settings, and that also demand time from nurses in application and from the nursing team in the execution of care actions made possible by the results of these scales. Therefore, it is necessary to think about a form of integration of the information generated, through the use of different scales/instruments present in nurses’ work and the PCS itself, in order to optimize care management and the best allocation of personnel, avoiding greater team overload.

To add to managers the ability to look at other NWL dimensions, a scale was developed and validated for the Brazilian reality containing factors and methods of work organization: available resources; team work; in-service education; care plan and follow-up; patient/family care; and meeting identified needs. The instrument can contribute, in a prospective way, to the detection of hazards, personnel planning, process improvement and recognition of the product delivered by nursing, and, combined with PCS, can assist in workforce planning^(30)^.

The scale referred to is anchored in the concept of complexity and adaptability in health work and centered on the nursing care process, on the relationship with patient/family and between professionals/services. In other words, it allows assessing critical points that make it impossible to deliver resilient and safe care or highlighting favorable scenarios for benchmarking. However, it is not proposed to measure the direct time dedicated to tasks, and therefore, its association with the use of a validated PCS is recommended^(30)^.

As verified by scoping review, methods that consider more factors tend to detect higher NWL^(25)^. Knowing the complexity of nursing work, it is inferred that this is natural and even expected, in addition to confirming that PCS, by themselves, cannot express the entirety of this “variable” that is the NWL. Thus, the human factors management model is highlighted, considering aspects related to the unit (patient/professional ratio and level of dependence of patients), work (perceived workload) and tasks (agility to perform, concentration mental, interruptions and others)^(26)^.

In Canada, in the analysis of the effects of these factors on patients and staff, perceived overload and task interruptions were found to be predictors of nurses’ ability to complete activities, implying omission of care^(26)^. The performance of multiple tasks by nurses that are not necessarily included in the daily assessment of a PCS was verified by another study^(28)^. Therefore, it appears that the work demand experienced by the nursing team needs to be accepted, that is, professionals need a space for explanation, debate and collective construction of actions to mitigate the overload, since the analysis of the rational elements for NWL measurement, in isolation, may not achieve full precision in the proposed assessment.

In addition to the activities mentioned, studies emphasize the inclusion of patient turnover, related to the number of admissions, discharges and transfers^(9,25)^ in NWL measurement, and the qualification of this process linked to the time dedicated by nursing^(9)^. Nurses also dedicate significant time to records and documentation in medical records^(29)^. Thus, it is inferred that the list of evidence on work activities not sensitive to PCS seems to be exponential, and this study aims to highlight this issue.

The challenge is set, in the sense of awakening dormant dimensions and still little discussed in health institutions. The multifactorial nature of NWL requires, in addition to assessing patients’ needs, an attentive look at the profile and perceptions of nursing and interprofessional team, recognition of particularities in the micro and macro institutional system and delineation of circumstances and tasks that affect overload and unsafe care.

## FINAL CONSIDERATIONS

The development of instruments capable of identifying nursing care needs is undoubtedly a landmark worthy of consideration by the profession, which deserves due recognition. Known as PCS, such instruments are fundamental in workforce planning and sizing, constituting, therefore, an indispensable tool in NWL rationalization, especially in hospitals.

On the other hand, the evolution of activities and the complexity of nursing practice environment have shown that the PCS are not sensitive to the completeness of the workload that professionals routinely face. This means that there are both objective and subjective nuances of this “variable” that go beyond verification mediated by a scale/instrument, even if it is highly qualified and duly validated.

The criticisms listed in this reflection point out PCS limitations, but do not intend to contraindicate their use, when properly validated and sensitive to the proposed assessment. However, the reflections put forward emphasize the need for further study and practice on NWL measurement, with consequent refinement of their instruments, so that they are able to cover the factors previously highlighted and others natural to emerge in the work evolution, i.e., to avoid NWL underestimation.

Due to NWL complexity and multidimensionality, it is important to consider that the thought of a single instrument to measure this entire construct is possibly impractical. In this way, it is considered with this study that the definition of this metric, especially when considered for workforce planning/sizing purposes, deserves to be complemented by elements in addition to PCS at the micro and macro institutional levels.

## ASSOCIATE EDITOR

Cristiane Helena Gallasch
